# Cisternal nicardipine for prevention of delayed cerebral ischemia in aneurysmal subarachnoid hemorrhage: a comparative retrospective cohort study

**DOI:** 10.1007/s00701-024-06023-z

**Published:** 2024-03-12

**Authors:** Alberto Vandenbulcke, Mahmoud Messerer, Marta Garvayo Navarro, David R. Peters, Daniele Starnoni, Lorenzo Giammattei, Nawfel Ben-Hamouda, Francesco Puccinelli, Guillaume Saliou, Giulia Cossu, Roy T. Daniel

**Affiliations:** 1 Department of Neurosurgery, University Hospital of Lausanne (CHUV), University of Lausanne, Rue du Bugnon 46, 1011 Lausanne, Vaud, Switzerland; 2 Department of Intensive Care, University Hospital of Lausanne (CHUV), University of Lausanne, Lausanne, Vaud, Switzerland; 3 Department of Radiology, Section of Neuroradiology, University Hospital of Lausanne (CHUV), Lausanne, Vaud, Switzerland

**Keywords:** Aneurysmal subarachnoid hemorrhage, Cerebral vasospasm, Cisternostomy, Delayed cerebral ischemia, Intrathecal nicardipine

## Abstract

**Purpose:**

Intrathecal vasoactive drugs have been proposed in patients with aneurysmal subarachnoid hemorrhage (aSAH) to manage cerebral vasospasm (CV). We analyzed the efficacy of intracisternal nicardipine compared to intraventricular administration to a control group (CG) to determine its impact on delayed cerebral ischemia (DCI) and functional outcomes. Secondary outcomes included the need for intra-arterial angioplasties and the safety profile.

**Methods:**

We performed a retrospective analysis of prospectively collected data of all adult patients admitted for a high modified Fisher grade aSAH between January 2015 and April 2022. All patients with significant radiological CV were included. Three groups of patients were defined based on the CV management: cisternal nicardipine (CN), ventricular nicardipine (VN), and no intrathecal nicardipine (control group).

**Results:**

Seventy patients met the inclusion criteria. Eleven patients received intracisternal nicardipine, 18 intraventricular nicardipine, and 41 belonged to the control group. No cases of DCI were observed in the CN group (*p* = 0.02). Patients with intracisternal nicardipine had a reduced number of intra-arterial angioplasties when compared to the control group (*p* = 0.03). The safety profile analysis showed no difference in complications across the three groups. Intrathecal (ventricular or cisternal) nicardipine therapy improved functional outcomes at 6 months (*p* = 0.04) when compared to the control group.

**Conclusion:**

Administration of intrathecal nicardipine for moderate to severe CV reduces the rate of DCI and improved long-term functional outcomes in patients with high modified Fisher grade aSAH. This study also showed a relative benefit of cisternal over intraventricular nicardipine, thereby reducing the number of angioplasties performed in the post-treatment phase. However, these preliminary results should be confirmed with future prospective studies.

## Introduction

Cerebral vasospasm (CV) is a major cause of mortality and morbidity in patients after aneurysmal subarachnoid hemorrhage (aSAH) [4, 8]. It is associated with delayed ischemic neurological deficit (DIND) and delayed cerebral ischemia (DCI)[45] in up to 30–40% of patients despite adequate treatment [26], and these events correlate with a worse neurological outcome [30, 33, 41].

CV management is based on prevention and treatment [26]. Prevention typically includes the administration of oral nimodipine and maintenance of euvolemia, while treatment is based on induced hypertension [11]; oral, intraarterial, and intravenous vasoactive drug administration [14, 20]; and mechanical angioplasty [8]. Intrathecal injections through a ventricular or lumbar drain have been proposed as an alternative method for the administration of vasoactive drugs, allowing higher concentrations of drugs in affected arteries while decreasing systemic side effects [31, 42]. Multiple studies have shown that prophylactic intraventricular and intracisternal nicardipine and milrinone are associated with a significant improvement in angiographic vasospasm, increase in mean cerebral blood flow, and a reduction in DCI rate, but their use did not show an improvement in the functional outcome for patients at high risk of CV [13, 19, 25, 26, 29, 39, 42, 47].

On the contrary, a recent study on the use of intraventricular nicardipine to treat significant CV confirmed its efficacy in DCI reduction and improvement of functional outcomes [36].

The impact of intracisternal injections of nicardipine through a cisternal drain (CD) as a treatment for moderate and severe vasospasm has never been investigated. The aim of this study is to investigate the effect of cisternal nicardipine in the treatment of moderate to severe CV. We compared patients treated with cisternal nicardipine to a similar cohort where nicardipine was delivered through an external ventricular drain (EVD) and to a control group where no intrathecal nicardipine was administered.

## Methods

### Patients and study design

We performed a single-center, retrospective analysis of prospectively collected data for all patients admitted for aSAH at our institution between January 2015 and April 2022. A head and neck CT angiography (CTa) scan was performed for all patients to confirm the diagnosis of ruptured intracranial aneurysm and to analyze the location and size of the aneurysm (two maximal diameters in millimeters). The modified Fisher scale was used to classify the entity of aSAH [15]. We included adult patients (> 18 years old) with a modified Fisher grade of III or IV, because of their increased risk of CV. Only patients who developed significant radiological CV (defined as moderate or severe vasospasm) and who survived more than 3 days were included in the analysis. The rational for this choice was that DCI most frequently appears in the period where vasospasm risk is higher. Follow-up was performed by a register neurosurgeon or interventional neuroradiologist depending on the aneurysm’s treatment modalities. Medical and surgical data were retrospectively extracted and reviewed; only patients with complete management and follow-up data were included. The study is reported according to the STROBE guidelines (Strengthening the Reporting of Observational Studies in Epidemiology) [46].

Clinical presentation on admission was classified according to the World Federation of Neurosurgical Societies (WFNS) [49]. We dichotomized it into good (WFNS I-III) and poor clinical status (WFNS IV-V).

We investigated the effect of cisternal nicardipine in the treatment of moderate to severe CV. Primary outcomes included the rate of DCI and functional outcome at 6 months compared to a similar cohort where we administered intraventricular nicardipine through an EVD and to a control group with CV but not treated with nicardipine. Secondary outcomes included the number of angioplasties performed in each group and the safety profile defined by rate of adverse events (infections and shunt rates).

Univariate comparisons were performed with a Fisher’s exact test for categorical variables, Kruskal–Wallis, and Mann–Whitney *U* tests. A *p* value < 0.05 was considered a significant difference. Subgroup analysis for nicardipine administrated through a CD or EVD was performed. Univariate analysis was performed to exclude confounding factors. The analyses were performed using the statistical software package STATA version 17 (College Station, TX, StataCorp LP).

### Patients’ management

All patients included in the analysis were treated according to the aSAH international guidelines [8] and received early aneurysm treatment within 48 h after rupture. The aneurysms were treated through microsurgical clipping or an endovascular procedure according to their location, morphology, presence of an intraparenchymal hematoma, patients’ clinical status, age, and comorbidities.

In 2016, a more extensive cisternostomy with lamina terminalis and Liliequist membrane opening, along with cisternal drainage positioning, was added to our institutional protocol for the treatment of aSAH as an adjuvant procedure to surgical clipping, as previously described [16]. Patients who underwent microsurgical clipping routinely received a cisternal drain independently from the presence of acute hydrocephalus. Continuous post-operative CSF drainage was routinely performed for at least 7 days. No EVD was used for these patients as the lamina terminalis was opened during cisternostomy, thus creating a direct communication between the ventricular system and the basal cisterns. Otherwise, EVD was placed only in patients with acute hydrocephalus (those treated by coiling) and for patients treated by microsurgical clipping before 2016.

Patients with a poor clinical status (WFNS score IV or V) received an intracranial pressure (ICP) monitoring device and were admitted in the intensive care unit (ICU). All patients received our institutional medical protocol to prevent CV for at least 21 days [8], including oral nimodipine administration, maintenance of euvolemia, and mean arterial pressure (MAP) ≥ 90 mmHg. Nimodipine was reduced if infusion rate of norepinephrine to maintain a MAP ≥ 90 mmHg exceeds 20 µg/min. Nimodipine dosage was recorded to avoid a possible treatment bias. It was dichotomized into “full dosage” or “reduced dose” with a cut-off at 180 mg per day.

A post-operative CTa was performed in all patients within 48 h after treatment and trans-cranial Doppler (TCD) was performed daily between day 3 and 21. A mean blood flow velocity (BFV) > 120 cm/s or increase > 50 cm/s within 24 h or a Lindegaard index > 3 were considered evocative for CV [27]. Cta and perfusion CT (PCT) were then repeated with the appearance of new neurologic symptoms or if TCD detected CV. Cta was reviewed by a registered neuroradiologist, and CV was classified as moderate or severe when the arterial narrowing was 50–75% or > 75%, respectively, compared to the baseline Cta, in at least two main arterial trunks [22]. End of CV was considered with either a normal Cta or after 2 consecutive days of normal TCD measurements. Medical management of moderate to severe CV consisted of induced hypertension (target MAP > 100/110 mmHg) and strict normovolemia. We performed endovascular angioplasty in cases of refractory symptomatic CV and decreased cerebral perfusion on perfusion CT, or CV non-responsive to previously listed measures. Chemical angioplasty was performed with the following technique: intra-arterial vasodilator infusion of a mixture of milrinone 2 to 3 mg and nimodipine 2 mg per large vessel at the neck (i.e., internal carotid artery or vertebral artery) through 30 min. In case of remaining focal severe vasospasm after intra-arterial infusion, we added mechanical angioplasty with balloon angioplasty or Comaneci® device (Rapid Medical; Yokneam; Israel).

### Intracisternal and intraventricular nicardipine injection

In September 2019, we added the injection of intracisternal or intraventricular nicardipine (through a CD or an EVD, respectively) as early adjuvant treatment for moderate and severe CV. Treatment decision was based on angiographic evaluation to promptly treat CV and potentially prevent its deleterious effects, while also avoiding clinical misdiagnosis in unconscious patients. The protocol consists of (1) withdrawal of 7 ml of CSF, (2) injection of 4 ml of 1 mg/ml of nicardipine solution, (3) injection of 3 ml of sterile saline solution, and (4) the drain is clamped for 30 min.

We started the treatment within 48 h from CV diagnosis (confirmed on Cta), and we performed it twice a day for a minimum of 5 days and until radiologic vasospasm resolution.

### Definition of clinical outcomes and follow-up

DCI is defined as a cerebral infarction visible on CT or MR scan within 6 weeks after aSAH and not present on the CT or MR scan performed between 24 and 48 h after aneurysm treatment. DCI cannot be attributable to other causes such as surgical clipping, endovascular treatment, ventricular catheter, or intraparenchymal hematoma [45].

The functional outcome was assessed by using the modified Ranking Scale (mRS) at 6 months and was dichotomized as “favorable” (mRS ≤ 2) and “unfavorable” (mRS ≥ 3).

Clinical symptoms and hemodynamic changes observed within 30 min from nicardipine injections for at least 2 consecutive administrations were recorded and considered adverse effects.

Drainage-related infection (DRI) was defined as a positive CSF culture associated with a ratio glycorrhachia/glycemia < 0.4, increasing CSF protein, or pleocytosis on serially collected samples [28].

## Results

### Patient population

**Fig. 1:**
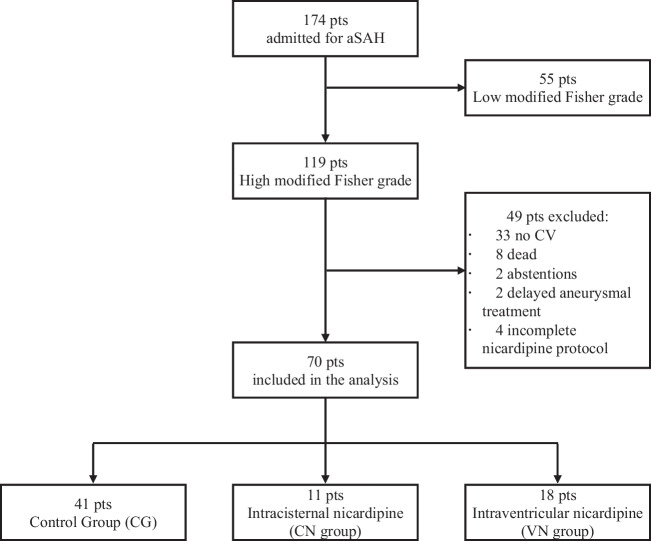
Flow chart showing the details of patient selection. aSAH, aneurysmal subarachnoid hemorrhage; CV, cerebral vasospasm; CG, control group; CN, cisternal nicardipine; VN, ventricular nicardipine

We identified 174 patients admitted for aSAH and 74 patients met the inclusion criteria. Patient selection is detailed in Fig. 1. A total of 70 patients were included in our analysis: 11 patients received intracisternal nicardipine (CN group), 18 patients received intraventricular nicardipine (VN group), and 41 patients with no nicardipine treatment were included in the control group.

Demographic and radiological characteristics of the population are reported in Table 1. Clinical and radiological conditions at admission were similar between the control group and the two intervention groups, but patients receiving intracisternal and intraventricular nicardipine were older when compared to the control group and with a trend towards more severe clinical and radiological presentation. A poor WFNS was reported respectively in 36% and 61% of patients receiving intracisternal or intraventricular nicardipine vs 29% in the control group, and a modified Fisher grade IV was reported in 73% and 78% of patients in the CN and VN groups vs 66% in the control group (Table 1). Globally, endovascular treatment was the most frequent technique used to secure the aneurysm (Table 2), but all the patients included in CN group had open craniotomy, aneurysm clipping, and CD placement, while in the VN group, endovascular procedures were used to secure the aneurysm and EVD was placed to treat acute hydrocephalus.

**Table 1:** Demographic characteristics of the subgroups: control group, cisternal nicardipine group, and ventricular nicardipine group

	CG	CN	VN
*N*°	41	11	18
Age mean, (SD)	51.8 (11.6)	56.9 (11)	58.6
Sex F:M	26:14	9:2	13:5
GCS at admission, mean (SD)	11.6 (4.,6)	11.4 (4.5)	9.9
WFNS I–III WFNS IV–V	28 (71%) 12 (29%)	7 (64%) 4 (36%)	7 (39%) 11 (61%)
Fisher III Fisher IV	14 (34%) 27 (66%)	3 (27%) 8 (73%)	4 (22%) 14 (78%)
Location			
MCA	11	9	1
ACA/AcomA	12	2	10
ICA	7		5
VA	2		
BT	2		1
PICA	3		

There were no significant differences between the three groups

*CG* control group, *CN* cisternal nicardipine group, *VN* ventricular nicardipine group, *SD* standard deviation, *F* female, *M* male, *GCS* Glasgow coma scale, *MCA* middle cerebral artery, *ACA* anterior cerebral artery, *AcomA* anterior communicating artery, *ICA* internal carotid artery, *VA* vertebral artery, *BT* basilar trunk, *PICA* posterior inferior cerebellar artery

**Table 2:** Treatment characteristics in the different subgroups: Statistically significant differences were obtained when comparing the control group with the cisternal and ventricular nicardipine groups for the DCI rate. Significant difference was observed only for the mean number of angioplasties between the control and the cisternal nicardipine group

	CG	CN	VN	*p*
*N*°	41	11	18	
Clip	10 (24%)	11 (100%)	0 (0%)	
Coil	31 (76%)	0 (0%)	18 (100%)	
Total drains EVD CD	27 20 (49%) 7 (17%)	11 0 (0%) 11 (100%)	18 18 (100%) 0 (100%)	
DCI	15 (37%)	0 (0%)	3 (17%)	0.02 (CG vs CN)
CV severity				
Moderate Severe	13 (32%) 28 (68%)	5(45%) 6 (55%)	6 (33%) 12 (67%)	
CV duration, mean ± SD	12.2 ± 5.8	10.4 ± 4.3	13.5 ± 4. 8	
Angioplasty, mean ± SD*	5.7* ± 3.9	2.1** ± 1.6	5.2*** ± 4.4	0.03 (CG vs CN)
CN treatment duration		9.2 ± 4		
VN treatment duration			10 ± 2.9	
Oral nimodipine dosage				
> 180 mg/die < 180 mg/die	15 (37%) 26 (63%)	6 (55%) 5 (45%)	11 (61%) 7 (39%)	

The mean number of angioplasties is reported among the patient who performed at least one: *Among *n* 22

^**^Among *n* 6

^***^Among *n* 12

*CG* control group, *VN* ventricular nicardipine, *CN* cisternal nicardipine, *EVD* external ventricular drain, *CD* cisternal drain, *CV* cerebral vasospasm, *DCI* delayed cerebral ischemia

An external drain was placed in all patients receiving nicardipine (11 CD and 18 EVD) and in 66% of cases in the control group (7 CD and 20 EVD). All patients received the standard protocol for management of CV, but 60% of the analyzed cohort needed a reduction of nimodipine dosage to maintain a MAP > 90 mmHg. No difference in terms of nimodipine administration (posology and duration of treatment) was observed between the three groups (Table 2).

We administered intracisternal or intraventricular nicardipine for a similar period in the CN and VN groups, with a mean of 9.2 (range 5–15) and 10 (range 5–18) days, respectively.

### Outcomes

**Fig. 2:**
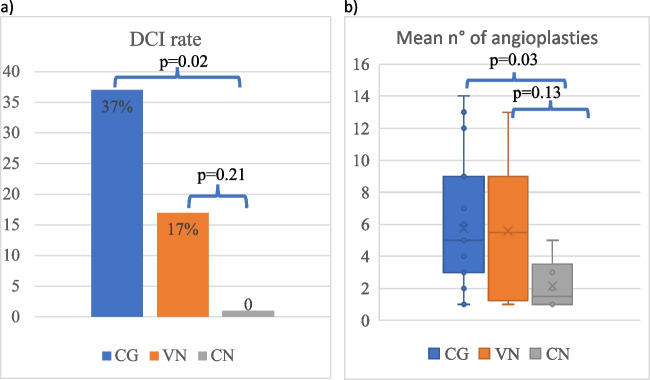
Comparison of DCI rate (**a**) and mean number of angioplasties per patient (**b**) between the control group (CG) and the two subgroups receiving intrathecal nicardipine (VN and CN). DCI, delayed cerebral ischemia; CG, control group; VN, ventricular nicardipine; CN, cisternal nicardipine

Intracisternal nicardipine was associated with a reduction in the incidence of DCI when compared to the control group (0% vs 37%, respectively, *p* = 0.02) (Fig. 2a). Cisternal nicardipine trended towards a further reduction in DCI rate when compared to the VN group but this difference was not statistically significant (0% CN group vs 17% in the VN group; *p* = 0.26) (Fig. 2a). None of the patients excluded from the analysis because of their low modified Fisher grade had DCI.

The use of a cisternal drain alone (with no nicardipine injection) was not associated with a reduction in DCI in the control group (*p* = 0.23).

**Fig. 3:**
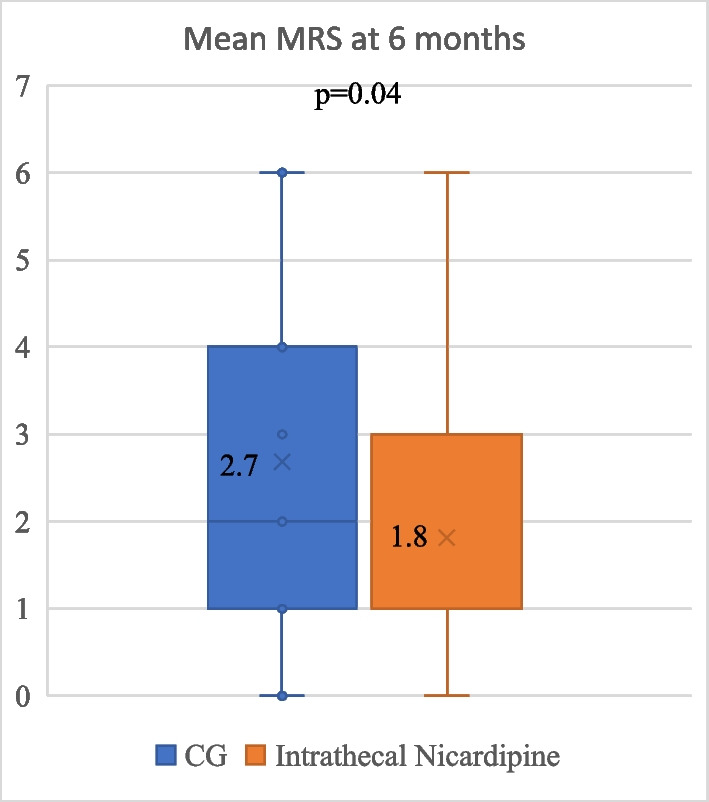
Comparison of mean mRS at 6 months between control and intrathecal nicardipine groups (VN and CN). CG, control group; VN, ventricular nicardipine; CN, cisternal nicardipine

Mean mRS at 6 months improved similarly in the cisternal and ventricular nicardipine groups compared to the control group (1.8 vs 2.7) but the difference was not statistically significant (*p* = 0.17 and *p* = 0.08, respectively). Favorable outcomes were recorded in 80% of patients in the cisternal group vs 54% in the control group (*p* = 0.16) (Table 3). There were no significant differences in functional outcomes between the CN group and the VN group (Table 3). Instead, if we consider all the patients receiving intrathecal nicardipine (either intracisternal or intraventricular), mean mRS at 6 months was significantly better than that of the control group (*p* = 0.04) (Fig. 3).

**Table 3:** Outcome characteristics: No significant difference was found in functional outcome between the three groups. The rate of hydrocephalus requiring a ventriculoperitoneal (VP) shunt was significantly higher in control group when compared to cisternal nicardipine group

	CG	CN	VN	***P***
mRS at 6 months, mean ± SD	2.7 ± 1.9	1.8 ± 1.8	1.8 ± 1.5	
mRS				
Good (0–2) Poor (3–6)	22 (54%) 19 (46%)	8 (80%) 2 (20%)	12 (71%) 5 (29%)	
Deaths	7 (17%)	1 (10%)	0 (0%)	
Infections	2 (7%)*	1 (10%)	1 (6%)	
VP shunt	11 (27%)	0 (0%)	8 (44%)	0.01 (CN vs VN)

The infection rate was calculated among the patients with external drains in the control group: *Among *n* 27

*CG* control group, *VN* ventricular nicardipine, *CN* cisternal nicardipine, *VP* ventriculoperitoneal

Intracisternal nicardipine was associated with a milder and shorter CV when compared to intraventricular nicardipine and to the control group but this difference was not significant. The period of treatment for the CN group and the VN group was similar (Table 3).

If we consider the number of intra-arterial angioplasties performed, 6 patients (55%) required an angioplasty in the CN group, with an average number of 2.1 sessions per patient. In the VN group, angioplasty was performed in 12 patients (67%), and the mean number of sessions for patients was 5.2. Cisternal nicardipine significantly reduced the mean number of sessions per patient (5.7 vs 2.1 *p* = 0.03) compared to the control group (Table 2a, b), and a similar trend was observed between VN and CN, but it did not reach a level of significance (5.2 vs 2.1, *p* = 0.13) (Fig. 2b). Comparing the VN group vs the control group, there was no significant reduction in the number of angioplasties performed (Table 2).

Among the entire cohort of patients, angioplasty was associated with an increased incidence of DCI (*p* = 0.01). Angioplasty was performed as a rescue treatment and DCI was more frequent in this group of patients. Among the entire cohort of patients, angioplasty was not associated with a reduction of DCI (*p* = 0.01). In the subgroup of patients receiving at least one angioplasty, patients also receiving intrathecal nicardipine experienced a significant reduction in DCI incidence (*p* = 0.02).

### Adverse events

Adverse effects related to cisternal or ventricular nicardipine administration were reported in 3 patients (2 in the CN group and 1 in the VN group), with a combination of headache, nausea, and vomiting. All three patients responded to supportive therapy (analgesia and antiemetic drugs) and received the full dosage. In one patient, the treatment was stopped because of two episodes of raised ICP after nicardipine administration.

No adverse systemic hemodynamic effects occurred.

No differences were found for drain-related infection rate between the three groups. None of the patients included in the CN group required a permanent shunt, while at the last follow-up, 44% and 27% of patients had shunt placement for chronic hydrocephalus in the VN group (*p* = 0.01) and CG (*p* = 0.09), respectively (Table 3).

## Discussion

Intrathecal administration of vasoactive agents in the management of cerebral vasospasm has been proposed as an alternative administration pathway to increase the local effects and avoid systemic hypotension [13, 25, 42]. This pathway seems to be associated with a more intense and durable angiographic response compared to intra-arterial injection in preclinical studies [31, 40].

Multiple injection pathways have been proposed, namely through external ventricular drains, cisternal drains, and lumbar drains. Prophylactic ventricular nicardipine has shown a potential benefit in reduction of clinical and radiological CV without significant clinical improvement [29, 47]. Prophylactic cisternal nicardipine decreased angiographic and symptomatic CV with a potential improvement of clinical outcome [40, 42]. Moreover, intra-operative placement of cisternal nicardipine prolonged-release implants significantly reduced the incidence of CV and DCI in severe aSAH [2, 23], while intraventricular implantation failed to reduce CV and DCI in coiled patients [3]. In contrast to a prophylactic or rescue approach, we explored an interventional approach, based on early angiographic diagnosis with a view to intervene following an early CV diagnosis in order to reduce the risk of DCI.

The use of intraventricular nicardipine in the treatment of moderate to severe vasospasm showed contrasting results, as it was associated with a significant reduction in mean cerebral flow velocity [29, 47], without significant outcome improvement in some preliminary studies [13, 29, 36, 47]. However, Sadan et al., instead, recently reported a significant DCI reduction and outcome improvement with ventricular nicardipine in a large retrospective study [36]. Cisternal nicardipine therapy has not yet been adequately explored. Suzuki et al. performed a prophylactic study on subarachnoid hemorrhage which showed benefits in incidence of vasospasm, though there were no comparisons with a control group [42]. Roelz et al. recently reported a cisternal lavage study for patients at high risk of DCI (implanted stereotactic ventriculo cisternal catheter) using fibrinolytic agents to reduce CV [34, 35]. They also instituted nimodipine therapy into the cisternal compartment when patients developed vasospasm to prevent DCI. The positive results for DCI and functional outcome in this study points towards the potential of cisternal therapies, even though there was no wide cisternal opening in this study. To our knowledge, our study is the first to explore the potential of an open cisternostomy and prolonged cisternal drainage and explore the superiority of cisternal administration pathway over the ventricular route. Cisternal nicardipine showed an enhanced vasoactive effect with an excellent safety profile. No cases of DCI occurred in the CN group (*p* = 0.02). The entire nicardipine group (intracisternal and intraventricular) was associated with a significantly lower rate of DCI (*p* = 0.01) and with an improved neurological outcome at 6 months (*p* = 0.04) when compared to standard medical treatment without nicardipine. However, this significance was lost when the intraventricular and the intracisternal group were considered separately, probably secondary to the small sample size of the individual groups. Furthermore, cisternal nicardipine significantly reduced the mean number of angioplasties when compared to the control group.

Ventricular nicardipine has previously been reported to have an increased risk of delayed hydrocephalus, without significant increase in risk of infection [36]. Chemical irritation and prolonged drainage may be responsible for chronic hydrocephalus [36]. In our cohort, a similar rate of definitive VP shunt placement was observed between the nicardipine group and the control group, while the cisternal group alone showed a significantly lower rate of shunts when compared to the ventricular group (*p* = 0.01) and control group (*p* = 0.09). Shunt-dependent chronic hydrocephalus was not the primary objective of our study, and the design of the study did not allow an exhaustive comparison between the groups. As we have previously reported, the performance of a cisternostomy and intraoperative washout of the basal cisterns seems to have a positive impact on shunt dependent hydrocephalus [16].

In our study, intracisternal and intraventricular nicardipine showed no significant direct effects on severity and duration of CV. There is growing evidence supporting the discrepancy between radiological vasospasm of large arteries, hypoperfusion, and DCI [10]. Multiple mechanisms of secondary injury may contribute to DCI, such as inflammation [12], microarterial constriction, and thromboembolism [9, 38, 44]. Intrathecal infusion of vasoactive agents might be more effective on both micro- and macrovascular CV [36] than systemic administration. Furthermore, compared to ventricular injections, cisternal drains deliver a high concentration drugs directly around the large proximal arteries [31, 42], leading to a high penetration in smooth muscle cells of the media [31, 32] and improved vasoactive responses [40, 42].

Moreover, open cisternostomy allows evacuation of subarachnoid blood in the basal cisterns, improving CSF circulation and intracranial pressure management while potentially decreasing CV and shunt rate [1, 43, 48]. Furthermore, there is potential benefit from continuous cisternal drainage post-operatively. This allows continuous washout of toxic blood products and improves control of intracranial pressure which may enhance nicardipine effects and increase cerebral perfusion, thus preventing DCI [18, 21, 37]. Emerging clinical data from the treatment of severe trauma brain injury shows the benefits of cisternal drainage in reducing the CSF shift oedema, marked improvement in management of intracranial hypertension and improved clinical outcomes [5–7, 17, 18]. The effect of these drains in aSAH treatment is likely to be due to similar mechanisms at play in its etiopathogenesis [43].

Intracisternal and intraventricular infusions allow high CSF concentration of drugs while maintaining low blood concentration, limiting unwanted systemic responses [24, 31, 42]. In our series, no cases of hypotension were reported after intrathecal injections. Headaches, though rare and typically benign, can occur immediately after administration and are thought to be induced by vasodilatations.

There are multiples limitations in this study. This is a retrospective analysis of a single-center series. This study had an unavoidable selection bias that was not negligible. In fact, nicardipine was introduced as an implementation of CV management for patients who already had an external drainage. This means that the baseline characteristics of the groups were by definition different. Patients who already had an external ventricular drainage were treated with ventricular nicardipine, while in the cisternal group, all the patients underwent surgical clipping, cisternostomy, and cisternal nicardipine treatment. Despite the fact that all study patients were treated based on institutional guidelines for aSAH management, there exist additional factors that can influence DCI occurrence and treatment that have not been controlled. Finally, the small sample size may underpower the statistical analysis and not adequately identify factors influencing the DCI, outcome, and the secondary outcomes investigated.

## Conclusions

Intrathecal nicardipine for moderate to severe CV seems to reduce the rate of DCI and improved long-term functional outcomes in patients with high modified Fisher grade aSAH. Cisternal administration showed a relative benefit over intra ventricular administration with no cases of DCI and significant reduction in the number of intra-arterial angioplasties performed in the post aneurysm exclusion phase of treatment. However, these preliminary results should be verified with future prospective studies with larger patient cohorts.

## Abbreviations

aSAH: Aneurysmal subarachnoid hemorrhage
CV: Cerebral vasospasm
CD: Cisternal drain
EVD: External ventricular drain
DCI: Delayed cerebral ischemia
CN: Cisternal nicardipine
VN: Ventricular nicardipine
DIND: Delayed ischemic neurological deficit
ICP: Intracranial pressure
ICU: Intensive care unit
CTa: CT angiography
TCD: Trans-cranial Doppler
BFV: Blood flow velocity
PCT: Perfusion CT
